# Coliform Bacteria as Indicators of Diarrheal Risk in Household Drinking Water: Systematic Review and Meta-Analysis

**DOI:** 10.1371/journal.pone.0107429

**Published:** 2014-09-24

**Authors:** Joshua S. Gruber, Ayse Ercumen, John M. Colford

**Affiliations:** Division of Epidemiology, University of California, Berkeley, California, United States of America; Wadsworth Center, United States of America

## Abstract

**Background:**

Current guidelines recommend the use of *Escherichia coli* (EC) or thermotolerant (“fecal”) coliforms (FC) as indicators of fecal contamination in drinking water. Despite their broad use as measures of water quality, there remains limited evidence for an association between EC or FC and diarrheal illness: a previous review found no evidence for a link between diarrhea and these indicators in household drinking water.

**Objectives:**

We conducted a systematic review and meta-analysis to update the results of the previous review with newly available evidence, to explore differences between EC and FC indicators, and to assess the quality of available evidence.

**Methods:**

We searched major databases using broad terms for household water quality and diarrhea. We extracted study characteristics and relative risks (RR) from relevant studies. We pooled RRs using random effects models with inverse variance weighting, and used standard methods to evaluate heterogeneity and publication bias.

**Results:**

We identified 20 relevant studies; 14 studies provided extractable results for meta-analysis. When combining all studies, we found no association between EC or FC and diarrhea (RR 1.26 [95% CI: 0.98, 1.63]). When analyzing EC and FC separately, we found evidence for an association between diarrhea and EC (RR: 1.54 [95% CI: 1.37, 1.74]) but not FC (RR: 1.07 [95% CI: 0.79, 1.45]). Across all studies, we identified several elements of study design and reporting (e.g., timing of outcome and exposure measurement, accounting for correlated outcomes) that could be improved upon in future studies that evaluate the association between drinking water contamination and health.

**Conclusions:**

Our findings, based on a review of the published literature, suggest that these two coliform groups have different associations with diarrhea in household drinking water. Our results support the use of EC as a fecal indicator in household drinking water.

## Introduction

Globally, drinking water has been established as a primary transmission pathway for diarrhea pathogens. [1], [2] In industrialized countries, centrally treated drinking water distribution systems have largely eliminated outbreaks of waterborne diseases, such as typhoid fever and cholera. [3] In developing countries, there is a large body of evidence that improving the microbial quality of drinking water by household treatment and safe storage reduces diarrhea. [4]–[6] Yet, evidence directly linking diarrheal illness to measured fecal contamination in drinking water remains inconclusive. [3], [7], [8]

In general, it is not feasible to test water for all known waterborne pathogens to assess whether it is safe for drinking. [2], [9]–[11] Instead, since the early 1900s there has been heavy reliance on fecal indicator organisms as measures of drinking water quality. [1], [12] Current World Health Organization (WHO) guidelines recommend *Escherichia coli* (EC) and/or thermotolerant (“fecal”) coliforms (FC) as indicators of the effectiveness of disinfection processes, and as index organisms for the potential presence of fecal contamination and waterborne pathogens; [2], [10], [12], [13] previous WHO guidelines use categories of EC and FC concentrations to define levels of disease risk from drinking water. [14] While EC are considered the most suitable indicator organism due to their specificity to fecal sources of contamination, FC are also recommended as an acceptable surrogate; this recommendation exists despite the recognition that the FC group includes coliform species of environmental origin, and is therefore not likely specific to fecal contamination. [2], [12], [13] Despite heavy reliance on EC and FC to assess the microbiological safety of drinking water, it has yet to be shown that either of these specific indicators is associated with waterborne illness. [8]

A previous systematic review and meta-analysis evaluated the evidence for the link between household drinking water quality, measured by fecal indicator organisms, and health; [8] the authors found no evidence of an association between diarrhea and indicators of drinking water contamination (EC, FC and *fecal streptococci*). This review, however, was limited by the availability, size and quality of articles published at the time the search was conducted (2001). [8] Specifically, the previous review included only three relatively small studies that reported using EC, and therefore focused mostly on FC as a measure of water quality. Given the limited number of studies available to the authors, they were also unable to evaluate the performances of EC and FC separately. Since the previous review, several studies have been published using EC as a measure of water quality (n = 6) [15]–[21], motivating our updated review with this newly available evidence. In addition, this larger body of evidence allows us to evaluate the performance of EC and FC separately; we hypothesized that the two coliform groups would have different associations with diarrhea given their different specificities for fecal contamination. Given the widespread use of EC and FC as measures of water quality by researchers and policy makers, and the limited evidence regarding the association of these proxy measures with actual disease outcomes, we believe that our systematic review and meta-analysis will make a significant contribution to the body of knowledge on the performance of indicator organisms in household drinking water, and have implications for their recommended use for future guidelines and research. In addition, based on the findings of our systematic review, we are able to make several recommendations for the design and reporting of future studies that assess the relationship between drinking water contamination and health.

## Methods

### Search Strategy

We searched MEDLINE, EMBASE, and Web of Knowledge databases for relevant articles. We used broad search terms for water, water quality and indicator organisms, diarrhea and household (point-of-use) sampling (Table 1). Titles and abstracts from the search were examined, and the full texts of relevant articles were reviewed. The bibliographies of relevant articles identified from full-text reviews were scanned to identify additional relevant studies. Searches were limited to articles published in peer-reviewed journals, in English, Spanish, German or Turkish (languages spoken by the authors). No restrictions were placed on date of publication. The final search was conducted on February 27, 2013. A protocol was not registered for this systematic review.

**Table 1:** Systematic Review Search Terms. a

Water Exposure	Household Sampling	Disease Outcome
water quality	household	Diarrhea
water microbiology	point of use	Diarrheoa
water pollution	Pou	gastrointestinal illness
water contamination	point-of-use	gastrointestinal disease
water supply	drinking water	gastrointestinal infection
drinking water	consumption	Dysentery
“indicator bacteria”	tap water	“HCGI”
“indicator organism”	well water	“highly credible gastrointestinal illness”
“thermotolerant coliforms”	domestic	“AGI”
“thermo-tolerant coliforms”		“AGII”
“fecal coliforms”		“acute gastrointestinal illness”
escherichia coli		
“e. coli”		
“fecal bacteria”		
“fecal contamination”		
“microbiological indicators”		

a Search terms combined using Boolean logic: within column terms combined using “or” statements; “and” statements were used to combine terms between columns.

### Inclusion and Exclusion Criteria

We reviewed articles that reported data on the association between exposure to *Escherichia coli* (EC) or thermotolerant (“fecal”) coliforms (FC) in household drinking water, and the occurrence of diarrhea under non-outbreak conditions; we excluded other, less commonly used indicator organisms (e.g., total coliforms, *fecal streptococci*). Our inclusion criterion was that the study collected exposure and outcome data at the household level or at the point of use; studies were excluded if they only reported measures of source water quality (e.g., shared wells, distribution system “nodes”), or used ecologic outcome data (e.g., health ministry reports). No further restrictions were placed on: definitions of diarrhea, recall period, study design, age groups, study location (developed/developing country), study setting (urban/rural), or drinking water source (as long as exposure to indicator bacteria was measured at the point of use).

### Study Reporting and Data Extraction

For each relevant study two authors (JG and AE) independently extracted basic study characteristics, including year of publication, sample size, age groups, study location, setting and design, indicator organism and enumeration methods, and diarrhea definition and enumeration methods into standardized forms, and resolved any discrepancies. Where possible, a relative risk (RR) such as odds ratio, cumulative incidence ratio, incidence density ratio or prevalence ratio was extracted from relevant studies. When no effect measure was reported by the authors, raw data were extracted to calculate an appropriate RR and confidence interval using standard methods. [22] If authors reported both raw data and unadjusted RRs we confirmed the reported estimates using the raw data; we used the RRs reported by the authors, unless otherwise noted. If authors reported results for both indicators (EC and FC) the results were treated as two separate studies in the meta-analysis. All RRs were extracted such that a value greater than unity indicated increased risk of illness among the group exposed to contaminated water. We also noted whether authors controlled for additional variables (by study design, stratified analysis or regression methods) and whether they accounted for correlation (clustering) of outcomes in their analysis.

### Exposure Thresholds

There are multiple ways to define comparison groups in water quality studies. For example, authors often use a threshold to define exposed and non-exposed groups based on indicator organism counts measured in 100 ml samples of water (i.e., exposed: ≥10 EC/100 ml; non-exposed: <10 EC/100 ml; with RRs calculated to compare these two groups); however, threshold levels can vary between studies. Current WHO guidelines recommend that drinking water that is safe for human consumption should have no detectable EC or FC in any 100 ml sample (i.e., a 1 EC/FC threshold); however, there is also evidence from the literature that much higher indicator organism concentrations are required to cause disease. For example, a study conducted in the Philippines on source water quality [23] previously reported a “threshold effect”, where no association with diarrhea was found at the 1 EC, 10 EC or 100 EC cutoffs, but an association was found at the 1000 EC cutoff; it is plausible that a similar threshold effect could be present for point-of-use drinking water. Given the limited number of studies included in the previously published systematic review and meta-analysis, the authors made no attempt to differentiate indicator organism-diarrhea associations at different threshold levels. [8] In our review, we attempted to extract RRs for different thresholds at the 1 EC or FC, 10 EC or FC, 100 EC or FC and 1000 EC or FC levels, when the authors reported these data.

### Categorical Exposures

As an alternative approach to exposure categorization, previous WHO guidelines defined disease risk from drinking water based on categories of indicator organism counts measured in 100 ml samples of water: 0 EC or FC, safe; 1–10 EC or FC, low risk; 11–100 EC or FC, intermediate risk; 101–1000 EC or FC, high risk;>1000 EC or FC, very high risk. [14] Investigators commonly use these guidelines to define exposure categories in studies of drinking water quality; each category is treated as an independent exposure group, and RRs are calculated for each elevated category relative to the 0 EC or FC exposure group. In our review, we extracted RRs for these categorical exposures to explore the presence of a dose-response (increased disease risk associated with increasing exposure levels) and to evaluate previous WHO guidelines that defined health risk based on these exposure categories. Within studies that evaluated dose-responses, we extracted *p*-values for tests of trend. In studies that did not report *p*-values for tests of trend but reported results for different categorical exposure levels, we extracted the appropriate data, where possible, and conducted our own tests of trend using previously published methods that estimate a log-linear dose-response while accounting for correlations between RRs from a single study (*glst* command: STATA 12, STATA Corp, College Station Texas). [24]

### Meta-analysis

For consistency with the previously published review, we conducted meta-analyses to calculate a summary measure pooled across all studies which provided an extractable RR, combining all EC and FC studies. [8] The previous review was not explicit about which results were used for meta-analyses if data could be extracted at multiple thresholds. In our review, we estimated a summary measure across all studies using the lowest extractable threshold from each study (e.g., if a study reported raw data that could be combined at the 1 EC, 10 EC and 100 EC thresholds, we included the RR calculated for the 1 EC threshold in the main meta-analysis). In addition, we conducted a sub-group analysis to explore the type of indicator organism used in each study as a source of heterogeneity in the main analysis; for this sub-group analysis we stratified studies by indicator used (EC and FC) and re-pooled summary measures separately for each indicator. In a secondary meta-analysis, we explored evidence for a threshold effect in household drinking water (see “Exposure Thresholds” above). For this analysis, we restricted meta-analyses to studies that reported extractable RRs at the 1 EC, 10 EC and 100 EC thresholds, respectively – this analysis was only feasible for studies that reported extractable data for all three threshold levels.

Summary measures were calculated by meta-analysis using random effects models with inverse variance weighting (STATA 12). [25] Heterogeneity across studies was evaluated using the Mantel-Haentzel χ^2^ test; we considered a *p*-value on the χ^2^ statistic <0.2 as evidence of heterogeneity. We used funnel plots to evaluate publication bias; plot asymmetry was interpreted as evidence of “small study bias”, for which publication bias is a likely contributor. [26], [27]

## Results

### Systematic Review

**Figure 1:**
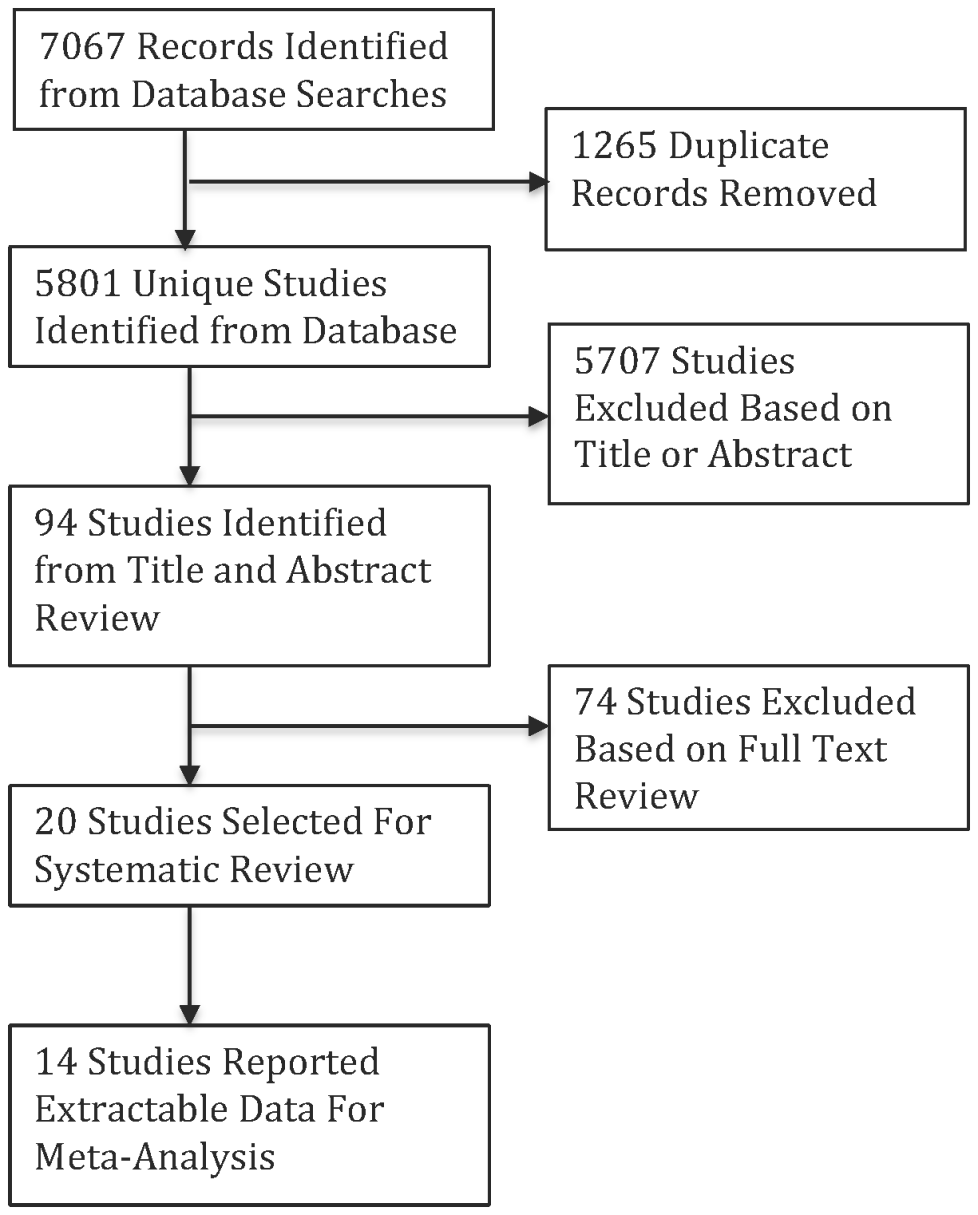
Systematic review flow chart.

Database searches were completed on February 27, 2013, from which we identified 5,801 titles and abstracts; 20 relevant articles were identified for review (Figure 1). Of the 20 relevant articles, 14 presented data in an extractable format (see Methods) and were included in meta-analyses. One article [28] presented results for both EC and FC; these results were treated as separate studies in the meta-analyses (resulting in 15 total studies).

### Study Characteristics: All Studies

The 20 relevant studies were published between 1977 and 2012 (Table 2). Two studies were conducted in a developed country (Canada), the rest in low-resource settings; no studies were conducted among populations with access to centralized water-treatment and distribution systems. The study settings included a mix of urban/peri-urban (n = 7) and rural (n = 13), and the study populations included a range of age groups (Table 2). The included articles used cohort (n = 14) and case-control (n = 6) study designs (Tables 3 and 4). We identified one randomized control trial [16] that reported data on the direct association between water quality and diarrhea; we considered this study a cohort design for the purposes of the review since water quality was not randomized. We note that, while cohort designs traditionally measure an exposure and then follow a population over time to measure outcomes, the cohort designs we identified in this review measured exposure (EC or FC): (i) simultaneously with outcomes through cross-sectional sampling; (ii) at intervals during ongoing diarrhea surveillance, or; (iii) the timing and analysis of water samples relative to diarrhea measurement was mixed or not clearly reported (Tables 3 and 4). The included case-control studies measured exposure after disease occurrence by design; however one study [19] utilized a nested case control design with risk set sampling in which disease and EC exposure were measured on the same day.

**Table 2:** Selected Characteristics of Included Studies.

First Author	Year	Location	Setting	Study Size^a^	Ages	Indicator
deAceituno	2012	Honduras	Rural	1020	All	EC
Levy	2012	Ecuador	Rural	115	NR	EC
Gundry	2009	S. Africa/Zimbabwe	Rural	254	1–2 y	EC
Brown	2008	Cambodia	Rural	1196	All	EC
Jensen	2004	Pakistan	Rural	209	<5 y	EC
Bhargava	2003	Bangladesh	Rural	99	1–10 y	FC
Strauss	2001	Canada	Rural	647	All	EC
Raina	1999	Canada	Rural	531	All	EC
Genthe	1997	S. Africa	Urban	316	“Pre-school”	Both
Jagals	1997	S. Africa	Urban	100	NR	FC
Vanderslice	1993	Philippines	Urban	254	<2 y	FC
Knight	1992	Malaysia	Rural	196	4–59 m	FC
Han	1991	Myanmar	Urban	208	6–29 m	FC
Henry	1990	Bangladesh	Rural	92	6–18 m	FC
Henry and Rahim	1990	Bangladesh	Urban	137	1–6 y	FC
Echeverria	1987	Thailand	Urban	NR	<5 y	FC^b^
Esrey	1986	Lesotho	Rural	545	1–60 m	FC
Lloyd-Evans	1984	Gambia	Urban	20	6–36 m	EC
Black	1982	Bangladesh	Rural	40	5–18 m	EC
Rajasekaran	1977	India	Rural	1091	<5 y	FC

EC: Escherichia Coli; FC: Fecal Coliform.

a May not reflect the analytic sample used by the authors;

b Authors report EC but methods suggest FC.

**Table 3:** Summary of EC Study Exposure and Outcome Measurements.

First Author	Year	Study Design	Outcome a	Recall	Measured EC Relative to Outcome	Enumeration Method	Medium	Temp (°C)^f^
deAceituno	2012	RCT	Diarrhea	7-day	After (<1 week)	MPN	IDEXX Colilert	NR
Levy	2012	CC	Diarrhea	Daily	Same Day	MF	BD Difco mI agar^e^ (EPA 1604)	30
Gundry	2009	Cohort	Diarrhea	Daily	Before (<2 weeks)	MPN	IDEXX Colilert	NR
Brown	2008	Cohort	Diarrhea	7-day	After (<1 week)	MF	“selective medium”^d^	NR
Jensen	2004	Cohort	Diarrhea	7-day	Before/After (<3 days)	MF	Hach m-ColiBlue 24 Broth PourRite	35
Strauss	2001	Cohort	AGII^b^	Monthly Diary	Twice over 28-d surveillance	MF	m-FC-BCIG agar	44.5
Raina	1999	Cohort	GI Illness^c^	Monthly Diary	5 times over 1-y surveillance	NR	“standard methods” (MOE 1992)	NR
Genthe	1997	CC	Diarrhea	Hospital	After	NR	“standard methods” (APHA 1989)	NR
Lloyd-Evans	1984	CC	Diarrhea	7-day	After (1–2 months)	MF	Millipore (DHSS 1969)	NR
Black	1982	Cohort	Diarrhea	NR	After (<1 month)	Plating	MacConkey's Agar	37

EC: *Escherichia Coli*; CC: Case Control; MF: Membrane Filtration; MPN: Most Probable Number; NR: Not Reported; C: Celsius; DHSS: Department of Health & Social Security: *The bacteriological examination of water* supplies; APHA: American Public Health Association: *Standard Methods for the Examination of Water and Wastewater*; MOE: *Ministry of Environment: Ontario's Drinking Water Objectives*.

a Unless noted, used a definition consistent with World Health Organization recommendation of 3 or more loose/watery stools in 24 hour period;

b Acute Gastrointestinal Illness: 1) vomiting or liquid diarrhea or 2) nausea or soft, loose diarrhea combined with abdominal cramps;

c Diarrhea with or with out vomiting;

d selective medium: “containing chromogenic and fluorogenic b-glucuronide and b-galactoside substrates”;

e Multiple EC enumeration methods used – we report the method with the largest sample size and for which the authors report an extractable RR;

f Incubation temperature.

**Table 4:** Summary of FC Study Exposure and Outcome Measurements.

First Author	Year	Study Design	Outcome^ a^	Recall	Measured FC Relative to Outcome	Enumeration Method	Medium	Temp (°C)^e^
Bhargava	2003	Cohort	GI Morbidity ^b^	1-month	After (<1 month)	MF	*DIFCO* m-FC media	45
Genthe	1997	CC	Diarrhea	Hospital	After	NR	“standard methods” (APHA 1989)	NR
Jagals	1997	CC	Diarrhea ^c^	Clinic	After	MF	“standard methods” (APHA 1992)	44.5
Vanderslice	1993	Cohort	Diarrhea ^c^	7-day	2–5 times over 1 year	MF	m-FC agar (APHA 1985)	44.5
Knight	1992	CC	Diarrhea	Clinic	After (4–14 days)	MF	*Paqualab* (ELE International 1986)	NR
Han	1991	Cohort	Diarrhea	7-day	Averaged over surveillance	MF	m-FC agar (WHO 1983)	NR
Henry	1990	Cohort	Diarrhea	7-day	Averaged over surveillance	NR	“standard methods” (APHA 1981)	NR
Henry and Rahim	1990	Cohort	Diarrhea	14-day	Unclear (<6 months)	dip-slides	*ORION Hygicult* Agar Slides ^d^	37
Echeverria	1987	CC	Diarrhea	Hospital	After	MF	m-FC agar (APHA 1975)	44
Esrey	1986	Cohort	Diarrhea ^c^	24-hour	Unclear	MF	Faecal Coliform Broth (AJPH 1975)	NR
Rajasekaran	1977	Cohort	Diarrhea	NR	Unclear (<15 days)	MPN	IMViC (APHA 1965)	44

FC: Fecal Coliform; CC: Case Control; GI: Gastrointestinal; MF: Membrane Filtration; MPN: Most Probable Number; NR: Not Reported; C: Celsius; APHA: American Public Health Association: *Standard Methods for the Examination of Water and Wastewater*; WHO: World Health Organization: *Guidelines for Drinking Water Quality*; IMViC: Indole, methyl red, Voges–Proskauer and citrate ^a a^ Unless otherwise noted, study used a definition consistent with current World Health Organization recommendation of 3 or more loose/watery stools in a 24 hour period; ^b^ Morbidity included: diarrhea, dysentery, vomiting, stomachache, acidity, typhoid, cholera; ^c^ Did not report a definition; ^d^ Report enumerating *Enterobacteriaceae* – included as FC for consistency with previous review. ^e^ Incubation temperature

Included studies measured EC (n = 9), FC (n = 10), or both (n = 1) in drinking water samples collected at the point of use. Overall, studies varied in their reporting of enumeration and incubation methods, and we could not always confirm the indicator organism enumerated based on the reported information (Tables 3 and 4). In at least one study, authors reported using EC, but a description of their methods suggested they measured FC. [29] With this exception, we relied on the authors' reporting or deferred to the categorization used by the previous review. [8]

Most studies used a definition of diarrhea that is consistent with current recommendations: three or more loose or watery stools in a 24-hour period (Tables 3 and 4). [30], [31] Four studies included the presence of blood or mucus in stool as part of their definition of diarrhea, [15], [17], [18], [32] and two studies [15], [17] reported dysentery as an outcome separate from “general” diarrhea; we did not include dysentery as a separate outcome in our review. Cohort studies in low-resource settings relied primarily on household surveillance and self-reported symptoms (e.g., maternal recall) for outcome assessment, using a range of recall periods (daily, up to one month; Tables 3 and 4); the two Canadian studies relied on monthly calendars maintained in the household and reported by telephone. Case-control studies mostly identified cases through hospitals and clinics, and selected controls from community samples; however, two studies identified cases and controls based on concurrent [19] or prior [33] household diarrhea surveillance conducted by the investigators.

Among the studies identified in our review, eight studies reported results that controlled for potential confounders; the other 12 studies found no evidence for confounding, or did not report attempting to control for confounding (Tables 5 and 6) – we did not differentiate between these studies in meta-analyses. In general, reporting on the independence of observations or methods to account for clustered outcomes was poor (Tables 5 and 6). Only five of the 20 studies reported using methods to account for correlated outcomes (e.g., generalized estimating equations; GEE). In general, the RRs and confidence intervals we calculated from available raw data were consistent with those reported by the authors, whether or not they reported accounting for correlated outcomes. However, in one of these studies, that reported using GEE, we calculated confidence intervals from extracted raw data that were substantially more conservative than those reported by the authors; [15] we included the more conservative confidence intervals for this study in our meta-analyses (Table 5).

**Table 5:** EC Study Results.

First Author	Year	Adjusted Analysis	RR	Threshold Results a RR (95% CI)	Categorical/Dose Response: bRR (95% CI)	Alternate Results: RR (95% CI)
deAceituno	2012	Yes	OR c	-	-	Log10 increase: 1.26 (1.08,1.46)
Levy	2012	NR	OR c	1000 EC: “no effect”	no “dose-response” (1–10 EC, 11–100 EC, 101–1,000 EC, 1,000+ EC)	Log10 increase: 1.29 (1.02,1.65)
Gundry	2009	Yes	NR	-	-	“no association”
Brown d	2008	No	PR c	1 EC: 1.55 (1.36,1.76) 10 EC: 1.70 (1.52,1.90) 100 EC: 1.67 (1.51,1.84) 1000 EC: 1.37 (1.21,1.54)	1–10 EC: 0.98 (0.81–1.20) 11–100 EC: 1.35 (1.13–1.61) 101–1000 EC: 1.83 (1.58–2.11) 1001+EC: 1.81 (1.54–2.11) e	-
Jensen	2004	No	IDR	1 EC: 1.32 (0.77,2.27) 10 EC: 1.25 (0.83,1.88) 100 EC: 1.23 (0.85,1.80)	1–10 EC: 1.17 (0.55–2.47) 11–100 EC: 1.26 (0.70–2.28) 100+EC: 1.45 (0.81–2.59) f	-
Strauss	2001	Yes	OR c	1 EC: 1.52 (0.33,6.92)	0.1–1.5 EC: 0.85 (0.10,7.19) 1.6–700 EC: 2.69 (0.34,21.56) g	-
Raina	1999	Yes	OR c	1 EC: 2.11 (0.90,4.94)	-	-
Genthe	1997	Yes	OR	1 EC: 1.26 (0.59,2.72)	-	-
Lloyd-Evans	1984	No	OR	1 EC: 3.86 0.33,45.57)	-	-
Black	1982	NR	NR	-	-	“no correlation found”

EC: *Escherichia Coli*; RR: Relative Risk; OR: Odds Ratio; PR: Prevalence Ratio; IDR: Incidence Density Ratio; NR: Not Reported; CI: Confidence Interval;

a Compares risk in groups exposed to ≥EC value, to <EC value;

b Reference group is <1 EC for all categories;

c Authors report using methods to account for correlated (clustered) outcomes;

d RRs and 95% CIs calculated from raw data;

e *p*-value for linear trend <0.01;

f p-value for linear trend  = 0.26;

g p-value for linear trend  = 0.45.

**Table 6:** FC Study Results.

First Author	Year	Adjusted Analysis	RR	Threshold Results a RR (95% CI)	Alternate Results RR (95% CI)
Bhargava	2003	Yes	NA	-	ML Coefficient: 0.204 (SE: 0.08) *p-value* <0.05
Genthe	1997	Yes	OR	1 FC: 0.84 (0.46,1.52)	
Jagals	1997	NR	NA	-	Geometric mean FC higher in homes with diarrhea (compared to control homes)
Vanderslice	1993	Yes	NA	-	Probit coefficient: −0.002 *p-value* >0.10
Knight	1992	Yes	OR	1 FC: 1.45 (0.57,4.76)	-
Han	1991	No	PR	Medium: 0.72 (0.56,0.94) b	Medium-High: 0.73 (0.52,1.02) c High: 0.72 (0.52,1.00) c
Henry	1990	No	IDR	Low: 1.03 (0.75,1.42) b	-
Henry and Rahim	1990	No	CIR	10^4^ FC: 1.26 (0.59,2.72) d	Improved Area: 2.58 (0.70,9.54) d Unimproved Area: 0.86 (0.34–2.18) d
Echeverria	1987	NR	NR	-	FC isolated from water as often in homes with diarrhea as in control homes
Esrey	1986	No	PR	10 FC: 1.53 (0.69,3.40) 100 FC: 1.46 (0.62,3.45)	10–100 FC: 1.41 (0.53,3.76) e>100 FC: 1.64 (0.64,4.18) e
Rajasekaran	1977	No	PR	10 FC: 2.93 (1.10,7.81)	-

FC: Fecal Coliform; RR: Relative Risk; OR: Odds Ratio; PR: Prevalence Ratio; IDR: Incidence Density Ratio; NR: Not Reported; CI: Confidence Interval; ML: maximum likelihood

a Compares risk in groups exposed to ≥FC value, to <FC value;

b Based on median of samples or arbitrary cutoffs;

c Compared to “Low” group – Authors do not report specific FC concentrations used to determined categories;

d Reference group is <1000 colony forming units FC per gram;

e Reference group <10 FC.

### Disease-Indicator Associations

Tables 5 and 6 summarize results from EC and FC studies, respectively. Among the ten EC studies, eight reported data that quantified the relationship between indicator exposure and diarrhea (Table 5). Of the eight studies with quantitative results, six reported threshold results at the 1 EC/100 ml cutoff [15], [18], [20], [21], [28], [33] and all six reported RRs greater than unity; however, only one study was able to rule random error out as an explanation for their results (Table 5). [15] The remaining two studies, among the eight that quantified a relationship between EC and diarrhea, reported significant relationships between diarrhea and linear increases in exposure to mean log10 EC concentrations; the results were very consistent across these two studies (Table 5). [16], [19] The two studies that did not have extractable, quantifiable results reported that no “association” or “correlation” was found between EC and diarrhea. [17], [34] However, we could not confirm whether the authors' conclusions were based on the size of the effect estimate (i.e., similar to unity), precision (i.e., “non-significant” confidence intervals or *p*-values), or both.

Among the 11 FC studies (including FC results from Genthe et al. 1997 as separate from their EC results), nine studies reported data that quantified the relationship between indicator exposure and diarrhea (Table 6). Results from these nine FC studies were less consistent than results from EC studies. Using the lowest extractable threshold, five studies reported estimates that suggest increases in the risk of diarrhea with exposure to FC [32], [35]–[38], and four reported effects equivalent to unity or reduced diarrhea risk with exposure to FC. [28], [39]–[41] Henry and Rahim (1990) reported FC effects for two distinct study areas differentiated by “adequate” versus “poor” sanitation infrastructure; while a single summary measure for this study suggested an increased risk of diarrhea with exposure to FC, the results from the two separate study populations were very different (Table 6; FC counts were associated with an increase in illness in the area with improved sanitation and a reduction in illness in the area with unimproved sanitation). Of the two FC studies that did not report extractable data, results were also mixed: one reported no difference in FC isolation from water in case and control households, [29] the other reported that households with diarrhea had higher overall geometric mean FC levels compared to control households. [42]

### Summary Measures

**Figure 2:**
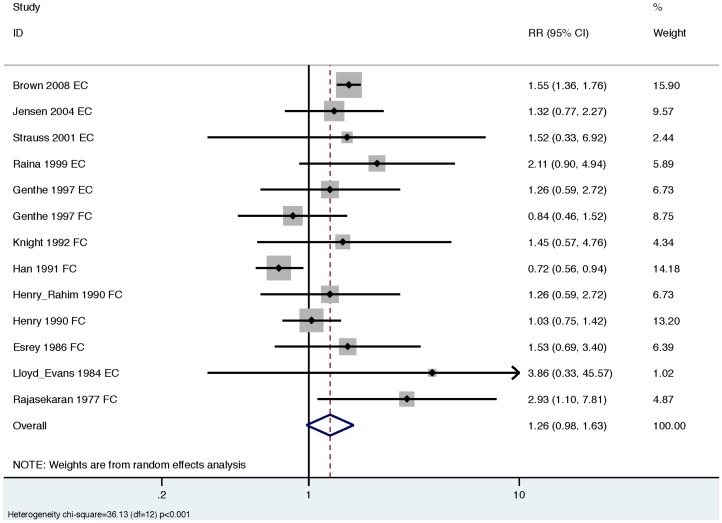
Forest plot for all included studies.

**Figure 3:**
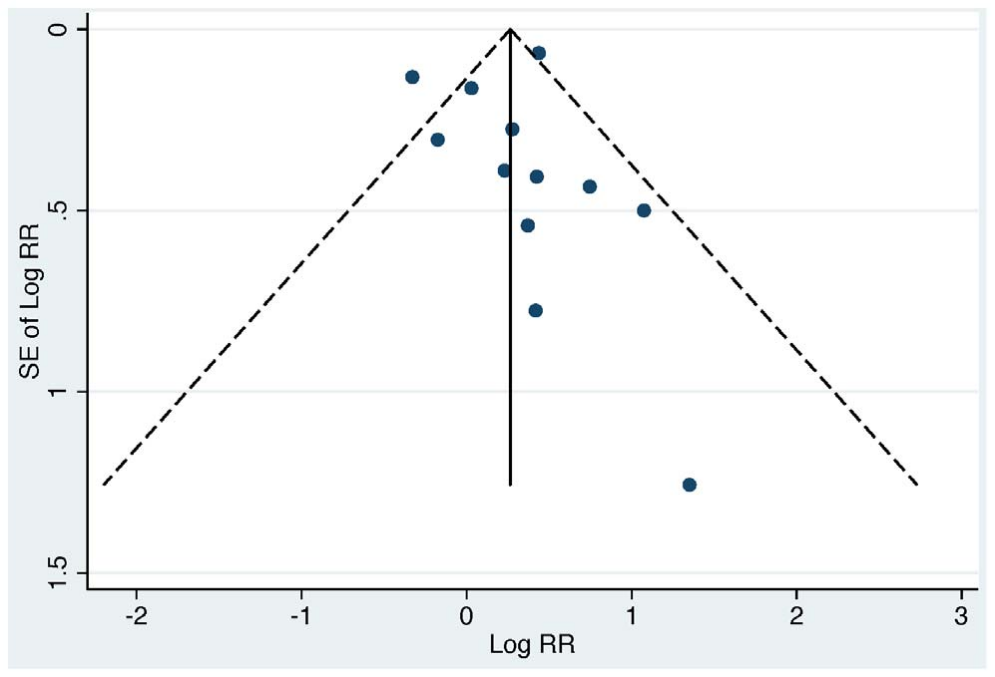
Funnel plot for all included studies (EC and FC studies). Studies are plotted relative to size (SE of Log RR), and reported log relative risk (Log RR); studies higher on the vertical axis are larger, and studies further to the right have larger RRs. The solid vertical line is the log of the summary RR for all studies, and the dotted lines are “pseudo 95% confidence intervals” for the summary measure. The absence of smaller studies with small or null RRs (lower left region of the plot) creates plot asymmetry, and provides evidence of possible publication bias.

For consistency with the previous review, we conducted meta-analyses across all studies (combining both EC and FC studies), using the lowest extractable threshold for each study (Tables 5 and 6). Across all studies, our summary estimate suggests a non-significant increase in diarrhea risk from exposure to drinking water contaminated with EC or FC (RR 1.26 [95% CI: 0.98, 1.63]; Figure 2). However, we also found evidence of significant heterogeneity (*Χ* ^2^ = 36.13, degrees of freedom (d.f.)  = 12, p<0.001), and evidence of publication bias (Figure 3).

**Figure 4:**
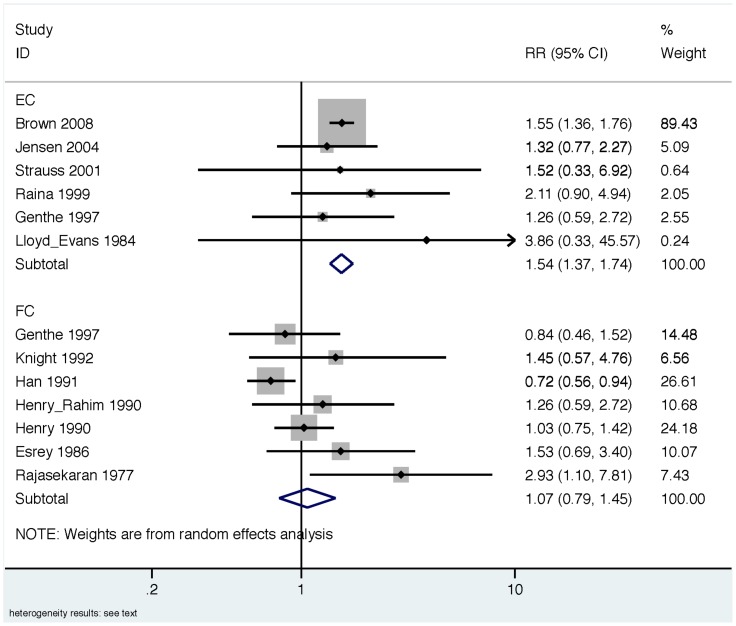
Forest plot for all studies stratified by indicator organism. Top panel, EC: *Escherichia coli*; Bottom panel, FC: thermotolerant (“fecal”) coliforms.

**Figure 5:**
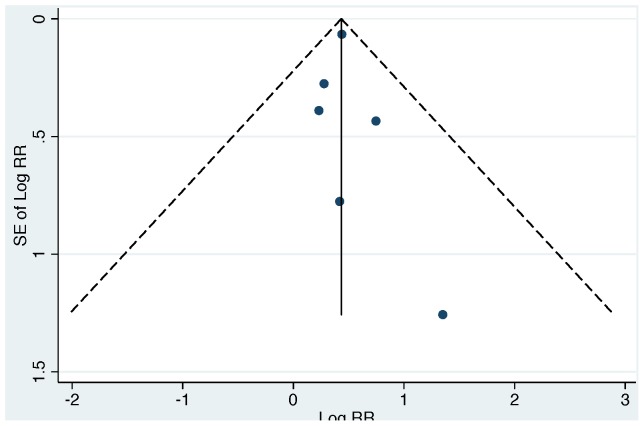
Funnel plot for EC studies. Studies are plotted relative to size (SE of Log RR), and reported log relative risk (Log RR); studies higher on the vertical axis are larger, and studies further to the right have larger RRs. The solid vertical line is the log of the summary RR for all studies, and the dotted lines are “pseudo 95% confidence intervals” for the summary measure. Studies on this plot are generally symmetrical, with the exception of one small study with a large RR, providing minimal evidence of publication bias.

**Figure 6:**
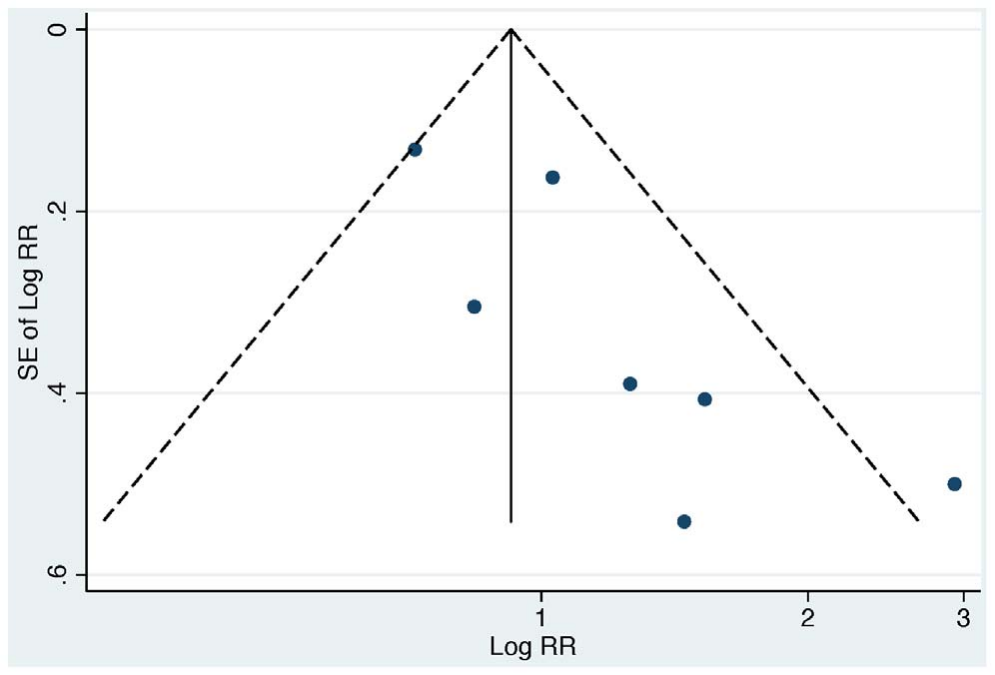
Funnel plot for all FC studies. Studies are plotted relative to size (SE of Log RR), and reported log relative risk (Log RR); studies higher on the vertical axis are larger, and studies further to the right have larger RRs. The solid vertical line is the log of the summary RR for all studies, and the dotted lines are “pseudo 95% confidence intervals” for the summary measure. The absence of smaller studies with small or null RRs (lower left region of the plot) creates plot asymmetry, and provides evidence of possible publication bias.

In order to explore a potential source of heterogeneity across all studies, we stratified results by indicator organism (EC vs. FC) and re-estimated summary measures in these sub-groups. Across all EC studies, the summary measure suggests a significant association between exposure to EC in household drinking water and diarrhea (RR: 1.54 [95% CI: 1.37, 1.74]); Figure 4). While we found no evidence of heterogeneity across EC studies (*Χ* ^2^ = 1.65, d.f. = 5, p = 0.90), and minimal evidence of publication bias (Figure 5), we note that the EC summary measure was disproportionately influenced by one study [15] (“% weight”, Figure 4). We therefore conducted a sensitivity analysis by repeating the EC analysis without the study by Brown et al. Removing this study did not impact the magnitude of the summary measure, but did reduce the precision of the estimate (RR 1.48 [95% CI: 1.02, 2.15]); Figure S1); there was no evidence of heterogeneity after removing Brown et al. from the analysis (*Χ* ^2^ = 1.59, d.f. = 4, p = 0.81). Across all FC studies, our summary effect measure suggests no association between FC exposure in drinking water and diarrhea (RR: 1.07 [95% CI: 0.79, 1.45]); Figure 4); we also found evidence of significant heterogeneity (*Χ* ^2^ = 2.30, d.f. = 6, p = 0.06), and publication bias (Figure 6).

### Threshold Effects

**Figure 7:**
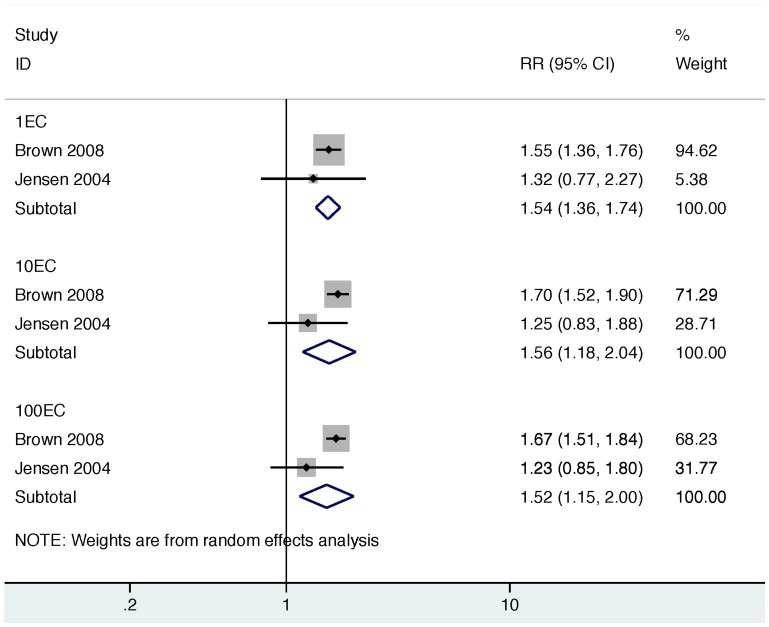
Forest plot for two studies with extractable data at multiple thresholds. Data were pooled across the two studies at the 1 EC/100 ml threshold (top panel), 10 EC/100 ml threshold (middle panel), and 100 EC/100 ml threshold (bottom panel). EC: *Escherichia coli*.

Two EC studies provided extractable data that allowed us to explore threshold effects at three different levels (1 EC, 10 EC, and 100 EC per 100 ml; Table 5) [15], [18]. RRs were calculated by comparing outcomes in the exposed group (≥EC threshold) to outcomes in the control group (<EC threshold). In one study, [15] increasing the threshold from 1EC to 10EC suggested a slight increase in diarrhea risk; there was no further increase in risk when the threshold was increased to 100EC (Table 5). In the other study, increasing the threshold beyond 1EC did not increase the magnitude of the effect estimates (Table 5). [18] We pooled estimates across these two studies, and found no evidence that increasing thresholds beyond WHO drinking water guidelines (1 EC/100 mL) was associated with an increased risk of diarrhea (Figure 7). [2] A third EC study reported finding no threshold effect at 1000 EC/100 ml, but did not report a RR. [19]

### Categorical Exposure and Dose-Response

Four EC studies reported investigating a dose-response relationship. [15], [18], [19], [21] Only three of these studies evaluated the presence of a dose-response using similar exposure categories (<1 EC (referent), 1–10 EC, 11–100 EC, 100+, or 101–1000 EC and 1000+; Table 5). Of these three studies, one did not report extractable data, but reported finding no evidence of a dose-response effect. [19] Results from the other two studies [15], [18] suggest a qualitative dose response (increased risk of diarrhea with increasing exposure categories; Table 5); formal tests of trend, however, had mixed results (Brown et al: p<0.001 provides evidence of a dose response; Jensen et al: p = 0.26 does not provide evidence of a dose response). A fourth study [21] used different EC exposure categorizations. The results from this study qualitatively suggested increasing risk across 0.1–1.5 EC and 1.6–700 EC exposure categories; however, the authors found no evidence of a linear trend (p = 0.45; Table 5).

## Discussion

### Summary of Findings

We conducted a systematic review and meta-analysis to evaluate the relationship between diarrheal illness and the presence of EC or FC indicators in household drinking water. Consistent with a prior review, [8] we did not find conclusive evidence for an association between contaminated drinking water and diarrhea when EC and FC results were combined (previous review: RR 1.12 [95% CI: 0.85, 1.48]; our review: RR 1.26, [95% CI: 0.98, 1.63]; Figure 2). We also found evidence of significant heterogeneity and publication bias across all studies (Figure 2, Figure 3). After stratifying studies by EC and FC to explore the choice of indicator organism as a source of heterogeneity, we found that EC studies reported consistent effect estimates that suggest an increased risk of diarrhea with exposure to contaminated drinking water with no evidence of heterogeneity (pooled RR 1.54 [95% CI: 1.37, 1.74]; Table 5, Figure 4). In contrast, FC studies reported mixed results with significant heterogeneity and provided minimal evidence of an association with diarrhea (pooled RR 1.07 [95% CI: 0.79, 1.45]; Table 6, Figure 4). We found no evidence that exposure thresholds greater than 1 EC/100 mL (e.g., 10 EC/100 mL, 100 EC/100 mL) were associated with increased diarrheal disease, but this evidence is based on a limited number of studies. Results regarding a dose-response relationship between EC in household drinking water and diarrhea were limited and inconclusive. In addition to the results of the meta-analysis, we identified several elements of study design and reporting that could improve future studies evaluating the relationship between water quality and health.

### Implications for Choice of Indicator Organism to Assess Microbiological Safety of Drinking Water

Current guidelines recommend two specific uses for indicator organisms: (i) fecal indicators (index organisms), used to indicate the presence of fecal contamination, potential pathogens, and possible health risk, and; (ii) process indicators used to evaluate the effectiveness of a water treatment process. [1], [2], [13]. While the performance of coliform bacteria as process indicators has been described previously, [2], [43] and is outside the scope of this review, the results from our review have implications for the use of EC and FC as index organisms in household drinking water. Currently, EC is considered the “most suitable” fecal indicator; however, FC is generally recognized as a suitable surrogate. [1], [2], [12], [13] Our findings support the continued use of EC as a fecal indicator (index organism) to assess water quality at the point of use, but do not support the use of FC as a surrogate as it does not demonstrate a clear association with diarrheal disease outcomes.

Our findings that EC and FC perform differently as indicators of diarrhea risk from contaminated drinking water are not surprising. While EC is generally of fecal origin, [10] and likely to be present simultaneously with diarrheagenic pathogens in recently contaminated drinking water, FC are known to include coliforms of environmental origin (e.g., *Klebsiella*), in addition to EC. [1], [2], [13] The consistency with which FC methods will be specific to human pathogens will therefore vary with the environments in which water samples are being collected. Indeed, our results show that the magnitude and direction of the effect estimates for FC were highly inconsistent (Figure 4, Table 6); even within a single study [37] the authors found highly divergent results using FC in two separate study areas (Table 6). In contrast, the magnitude and direction of effect estimates across the EC studies included in our review were consistent across diverse study regions, and when pooled provided evidence of a significant association with diarrhea; these results are consistent with the higher specificity of EC for fecal contamination, compared to FC (Table 5, Figure 4). The presence of a dose-response between EC and diarrhea could have provided further evidence to support our findings that drinking water contaminated with EC is associated with diarrhea. However, dose-response results from our review were inconclusive, and represent an avenue for future research.

While our summary measures suggest that the presence of EC in household drinking water is significantly associated with diarrhea, it is important to note that only one individual EC study in our review was able to rule out chance as a possible explanation of their findings [15]. It has been demonstrated that indicator levels can vary considerably even over short periods of time in both household and source water, [44], [45] and that pathogens and indicator organisms have inconsistent correlations. [46] The studies in this review relied on intermittent grab samples to classify household water quality. Even daily grab samples likely provided only a crude assessment of a household's exposure to fecal contamination and diarrheagenic pathogens, which likely contributed to the lack of precision in the individual studies included in our review. While the imprecision of results from individual studies supports a previous assertion that fecal indicator organisms are “blunt” tools to characterize water quality, [19] our findings suggest that they have continued utility in health and water research. Indeed, while recent WHO drinking water guidelines have shifted focus from recommendations using specific indicator levels to define water safety to a more integrated approach centered on water safety plans, these recommendations are largely directed towards the management of water distribution systems. [2], [13] In practice, regional organizations and researchers working in areas without access to drinking water distribution systems rely heavily on indicator organisms to assess the risk of disease from drinking water, and to measure the effectiveness of household water treatment and safe storage interventions; this is particularly true in resource poor settings with limited access to advanced laboratory facilities. Our results suggest that EC have continued value in both of these applications.

### Threshold Effects for Diarrhea Risk

The findings from our review do not support the existence of a “threshold effect” in contaminated household drinking water. A previous study evaluating the effect of contaminated *source* water on diarrhea [23] found no evidence of an association between EC and diarrhea at the 1 EC threshold level; however, significant associations with diarrhea were observed at the 1000 EC threshold level. The authors concluded that their findings of a “threshold effect” supported earlier assertions that relaxing drinking water guidelines (beyond the 1 EC threshold) might be acceptable in developing countries. [47] Contrary to these findings, in our review of household drinking water we found consistently elevated risks of diarrhea at the 1 EC threshold, with a significant summary measure when pooled across all studies. These results support current WHO guidelines recommending that all drinking water intended for human consumption contain zero EC/100 ml, regardless of location. [2] Beyond the 1 EC threshold, our results did not suggest increased risk of diarrhea at elevated cutoffs, but this evidence was based on a limited number of studies.

### Recommendations for Future Water Quality Studies

The studies included in our review covered several decades, and varied considerably with respect to methodological quality and completeness of reporting. In this section, we highlight several findings from our systematic review that could improve the design and reporting of future studies that seek to evaluate the relationship between water quality and health.

The studies in our systematic review used very consistent definitions of diarrhea, relying primarily on self- or caregiver reporting of diarrhea symptoms (Tables 3 and 4). However, we note that diarrhea recall periods and identification strategies varied greatly (e.g., daily recall, one-month recall, cases presenting to clinics; Tables 3 and 4). Reliance on self-reported diarrhea symptoms as a health measure could have introduced bias into individual studies through two primary mechanisms, which have implications for the use of self-reported diarrhea as an outcome in future studies. First, as with any self-reported, subjective outcome, there is a potential for differential reporting relative to exposure status [48]. If study-specific reporting biases were differential with respect to drinking water contamination, it is possible that the results of individual studies (and therefore our review) could be biased. However, there is no reason to suspect that individuals would be aware of the indicator status of their drinking water when reporting diarrhea symptoms, which would decrease the potential for this bias. Second, longer follow-up periods can bias diarrhea reporting due to poor outcome recall, which could have biased findings towards the null if non-differential with respect to exposure, and/or compounded biases associated with differential reporting in individual studies. [49] The inclusion of objective health outcome measures (e.g., pathogen-specific antibody responses) could improve future studies by reducing reliance on self-reported diarrhea symptoms and their associated biases. [50], [51] In addition, while our review focused exclusively on diarrhea, expanding the range of water-related health outcomes studied and reviewed (e.g., indicators of growth and malnutrition) could also improve our understanding of the health impacts of drinking water contamination beyond gastrointestinal illnesses.

Very few studies in our review were able to establish temporality between exposures (indicator organism) and outcomes (diarrhea), and therefore mostly relied on the assumption that drinking water samples were representative of an exposure period that was relevant to measured disease outcomes. While this assumption might be reasonable, or the only option given the practicalities of field conditions, it does leave open the possibility of reverse causality (i.e., diarrhea caused by other environmental pathways led to increased fecal contamination of drinking water). The design of future water quality studies should attempt to measure exposure in advance of disease outcomes, and all studies should be explicit about the timing of exposure and disease measurements in their reporting. These recommendations would all but preclude the use of “traditional” case-control studies, where cases and controls (i.e., disease status) are identified first, followed by visits to households to collect water samples to ascertain exposure status. Instead, a cohort design that measures exposure to contaminated drinking water first, followed by surveillance visits after an appropriate incubation period to measure health outcomes, is a more suitable study design. Decisions regarding follow-up frequency, recall period, and measures of disease occurrence (i.e., incidence, prevalence) will vary depending on study context and logistics, but should be carefully considered and explicitly reported. [49], [52]

In studies evaluating the relationship between drinking water quality and health, confounding bias is a concern: there are numerous factors that could cause both drinking water contamination and diarrhea in a given study setting. Overall, the studies in our review were not consistent in reporting adjusted results (Tables 5 and 6), and there was no consistent set of factors used across studies when authors did report attempting to control for confounding. Incomplete control of confounding in the included studies could have biased individual study results, and therefore the findings of our review. Future studies should be explicit about their attempts to measure and control for confounding factors. Additionally, several studies did not account for correlated outcomes in their analyses, which could have influenced the precision of summary estimates. However, for EC studies we note the consistency of the direction and magnitude of the associations reported across diverse study locations, and across studies that did and did not adjust for confounding and/or account for clustering. Regardless, reporting on these issues could be improved upon in future studies.

Our systematic review included published, peer-reviewed studies. As with any systematic review, publication bias, where smaller studies with small or null (“non-positive”) results are systematically excluded from the published literature, could have impacted our findings. In our review, we found evidence of publication bias among FC studies (smaller studies with smaller effect sizes appear to be absent from the funnel plot; lower left region, Figure 6); however, inclusion of smaller FC studies with RRs at or less than unity would have strengthened our findings of no association between FC and diarrhea. We found minimal evidence to suggest publication bias among EC studies (Figure 5); however, we cannot rule out publication bias as a possible explanation of our findings – the funnel plot suggests that most studies were balanced around the summary measure with the exception of one small study with a large positive finding. Taken together, there is evidence that smaller studies with small (“non-positive”) effects sizes are systematically missing from the literature on the relationship between water quality and diarrheal illness.

In our review, we pooled RRs across relevant studies using meta-analysis to investigate the association of fecal indicator measures in drinking water with diarrhea. The limitations of meta-analysis have been discussed [53]; we caution against interpreting the summary measures from our review as “true” underlying causal effects, but rather consider them additional pieces of evidence regarding the relationship between fecal indicators in drinking water and diarrhea. The evidence from our pooled summary measures, taken together with biologic plausibility and the consistency of results across studies, provides evidence to support a relationship between diarrhea and the presence of EC in point-of-use drinking water; we do not, however, find evidence to support an equivalent relationship between diarrhea and FC.

### Conclusions

We reviewed the available literature to identify studies that evaluated the relationship between microbial indicators of drinking water quality and diarrhea. We found that studies using *Escherichia coli* (EC) as an indicator of household drinking water quality reported consistent effect estimates, that when pooled suggested a significant association with increased diarrheal illness. Results from studies using thermotolerant (“fecal”) coliforms (FC), on the other hand, were inconsistent, and suggested no association with diarrhea when pooled. In this review, we also note several areas where the design and reporting of the included studies could have been improved, and make recommendations for future studies. The results from our review suggest that EC has value as a fecal indicator organism, but that use of FC should be considered carefully in contexts where an association with diarrheal disease outcomes is important.

## Supporting Information

Figure S1 **Forest plot of EC Studies Excluding Brown et al.**
(PDF)

Checklist S1 **PRISMA Checklist for Systematic Reviews and Meta-Analyses.**
(PDF)
