# Supplementary figures and images for: Coliform Bacteria as Indicators of Diarrheal Risk in Household Drinking Water: Systematic Review and Meta-Analysis
Source: PLoS One. 2014 Sep 24;9(9):e107429. doi: 10.1371/journal.pone.0107429 (PMC4175079; doi:10.1371/journal.pone.0107429)

Figure S1: Forest Plot of EC Studies Excluding Brown et. al.

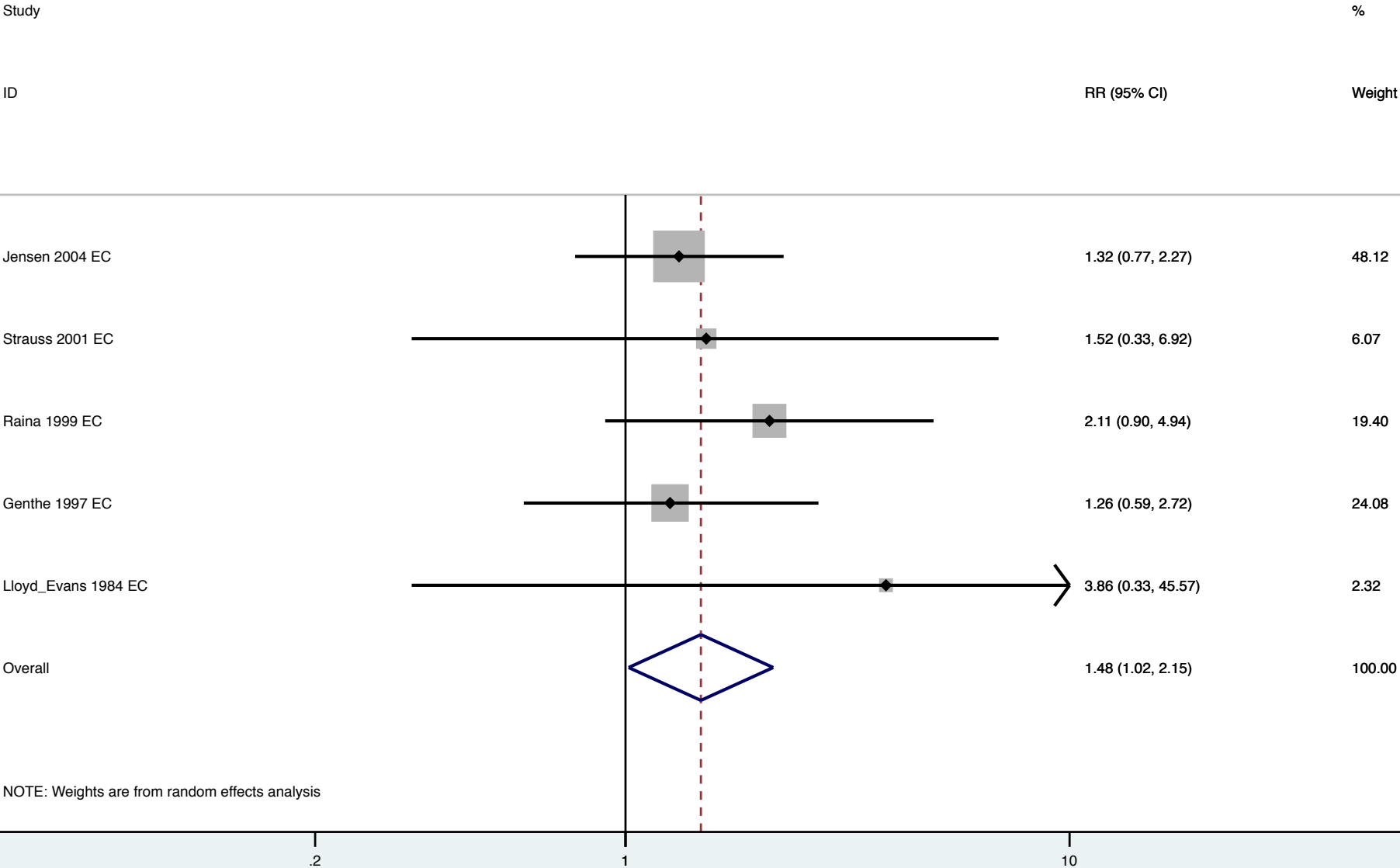

Supplement: Figure S1 — Forest plot of EC Studies Excluding Brown et al. (PDF) [file pone.0107429.s001.pdf]
